# Cannabis Use and Associated Gastrointestinal Disorders: A Literature Review

**DOI:** 10.7759/cureus.41825

**Published:** 2023-07-13

**Authors:** Adedeji O Adenusi, Hezborn M Magacha, Chisom M Nwaneki, Olamide A Asifat, Eugene N Annor

**Affiliations:** 1 Internal Medicine, Interfaith Medical Center, Brooklyn, USA; 2 Internal Medicine, East Tennessee State University, Johnson City, USA; 3 Internal Medicine, Saint Peter’s University Hospital, New Jersey, USA; 4 Epidemiology and Biostatistics, East Tennessee State University, Johnson City, USA; 5 Internal Medicine, University of Illinois, Chicago, USA

**Keywords:** cannabinoids, acute pancreatitis, peptic ulcer diseases, gastroesophageal reflux disorder (gerd), cannabinoid receptor 2, cannabinoid receptor 1, cannabis use

## Abstract

Cannabis, commonly known as marijuana, is used by at least 18% of the United States (US) population, which makes it the most commonly used federally illegal drug in the United States. It is widely used for recreational purposes, while its therapeutic benefits have been extensively explored in the US. For several years, cannabis has been used for the treatment of diverse health conditions, including pain management, anti-inflammatory effects, and spasticity associated with multiple sclerosis and other neurodegenerative diseases. However, cannabis use has been associated with some acute and chronic adverse effects. This review sheds light on gastrointestinal disorders, gastroesophageal reflux disease, pancreatitis, and peptic ulcer disease that have been associated with cannabis use.

## Introduction and background

Cannabis, also known as marijuana, has a long history of use in the United States, with evidence of its cultivation dating back to the colonial era [1]. As of 2019, it remains the most used federally illegal drug in the US, with about 18% (48.2 million) of Americans using it [2,3]. Despite its prohibition, cannabis use continued to be prevalent in the United States, particularly among young people [4]. Recently, attitudes toward cannabis use have shifted, and many states have sought to legalize its use for medical and/or recreational purposes. While it has remained illegal at the federal level, 36 states and the District of Columbia have, as of 2021, legalized cannabis for medical use, and 15 states and the District of Columbia have legalized cannabis for recreational use [5,6]. In addition to legal issues, cannabis use raises concerns about potential health effects, particularly for young people. According to the American Medical Association (AMA), the use of cannabis during adolescence is associated with negative health outcomes, including lower academic achievement, impaired memory, and an increased risk of gastrointestinal disorders [7].

However, cannabis has been identified as having therapeutic benefits due to its anti-inflammatory, anti-apoptotic, analgesic, antioxidant, neuroprotective, and neuro-modulatory effects [2]. For instance, in Oregon and Colorado, medical cannabis is used to manage chronic pain, post-traumatic stress disorder, epilepsy, glaucoma, multiple sclerosis-associated spasticity, and other neurogenerative diseases [8]. Nonetheless, clinical trials have suggested that the medical use of cannabis and other cannabinoid-based formulations is not supported by good clinical data [8]. Additionally, cannabis, when used therapeutically, has been associated with acute side effects such as impaired coordination, hyperemesis syndrome, suicidal ideations, and anxiety [3]. Chronic effects associated with cannabis use include cannabis withdrawal syndrome, neurocognitive impairment, respiratory diseases, cardiovascular diseases, and gastrointestinal diseases [5-9]. However, the association between cannabis and gastrointestinal disorders has not been extensively studied. Research in this aspect showed that therapeutic or recreational use of cannabis is associated with specific gastrointestinal disorders such as gastro-esophageal reflux disease (GERD), pancreatitis, and peptic ulcer disease. Our study aims to explore these gastrointestinal disorders in association with cannabis use in patients.

## Review

Gastroesophageal reflux disease

Gastroesophageal reflux disease (GERD), also known as acid reflux, is a condition where stomach acid flows back into the esophagus, causing symptoms such as heartburn, regurgitation, and discomfort. While cannabis use has been reported to have various effects on the body, including the digestive system, the relationship between cannabis use and reflux disease is not entirely clear. Some studies have suggested a potential association between cannabis use and an increased risk of GERD symptoms, while others have reported conflicting results or no significant association. A 12-year retrospective case-control study done by Meet Parikh et al. documented that chronic cannabis users were more likely to complain of heartburn compared to their controls (non-cannabis users) [10]. Furthermore, a case report by Joseph Levy et al. in 2020 documented endoscopic findings suggestive of barrette esophagus in a patient with chronic cannabis use [11]. In the United States, GERD has a prevalence of 18.1%-27.8% [12], and the possible contributions of cannabis use to GERD-associated disorders cannot be overemphasized.

Transient lower esophageal sphincter relaxation (TLESR) is the most common mechanism underlying GERD, normal lower esophageal relaxations are associated with swallowing, whereas TLESRs are unrelated to swallowing [9,11]. The lower esophageal sphincter is a muscular ring that separates the esophagus from the stomach and helps prevent stomach acid from flowing back up. Some studies suggest that cannabis use may relax the LES, which could lead to an increased risk of acid reflux. A study done by Parikh M., Sookal S. et al. revealed that cannabis acts on receptors in the lower esophagus to increase TLESRs and thereby increase the risk of GERD [10]. Smoking cannabis, particularly if inhaled deeply, can cause coughing and irritation of the airways. Chronic coughing and irritation may increase the likelihood of acid reflux by putting extra pressure on the LES and disrupting the normal functioning of the esophagus.

Two types of cannabinoid receptors have been identified in humans: CBR1 and CBR2. The principal CBR expressed in the GI tract is CBR1 [13]. Consumption of cannabis or administration of cannabinoid receptor (CBR) agonists has been shown to decrease the resting lower esophageal sphincter (LES) tone, while CBR antagonists have the opposite effect on LES tone. [14-16]. Furthermore, studies have shown that CB1 is highly expressed in patients with non-erosive esophageal reflux disease compared to healthy patients [17].

There are many controversies related to the causal effect of cannabis on GERD. In some studies, CB1-agonists reduced the TLESR (improved GERD symptoms), while in other studies, they reduced the LES (potentiated GERD symptoms) [18]. However, a placebo-controlled study done by Beaumont H., Jensen J., et al. in 2008 found a significant causal effect of cannabis, which reduced the LES pressure and triggered TLESR in humans [9]. Despite these controversies, TLESR remains a potential target for the pharmacological treatment of GERD.

While managing and treating GERD with acid-suppression therapy in patients, medical professionals must be aware that cannabis use could present with GERD. Given the overall increase in consumption of cannabis worldwide, physicians should be aware of this side effect and counsel all patients with intractable GERD to avoid the use of cannabis for any purpose.

Pancreatitis

Acute pancreatitis (AP) is one of the leading gastrointestinal causes of hospitalization in the United States [18]. Common causes of AP include alcohol, gallstones, medications, infection, trauma, and autoimmune disease. The clinical spectrum of this common disorder ranges from mild epigastric pain to circulatory shock and the resultant multi-organ dysfunction syndrome. Overall, it has a mortality rate of 1%-5%, which has improved over the years [10]. Over the last few decades, the incidence of AP and resultant hospitalizations have steadily increased, while at the same time, the prevalence of alcohol use has decreased [19]. This increased incidence, in part, is presumed to be related to gallstone disease, given the increased prevalence of obesity. Another major reason for the increased incidence is related to increased laboratory testing and frequent abdominal imaging [20,21]. Despite the advanced imaging and diagnostic modalities for diagnosing pancreatitis, approximately 20% of the diagnosed cases are classified as idiopathic. Cannabis use has been postulated to be associated with AP and is emerging today as an overlooked cause of idiopathic acute pancreatitis [22].

The mechanism by which cannabis causes acute pancreatitis is not clearly understood, but different studies postulate that it acts via CBR1 and CBR2, which are expressed in pancreatic tissues [23]. The interaction with these receptors results in the release of pancreatic enzymes such as lipase, amylase, and ribonuclease. Another hypothesis, according to Sagaram Manasa, Sundar, Preeyanka, et al., suggests that cannabis results in the relaxation of the sphincter of Oddi, which acts as a trigger for pancreatitis [11]. 

Our literature review revealed a total of 26 cases of cannabis-induced pancreatitis thus far. Some studies have linked cannabis use with an increased risk of post-endoscopic retrograde cholangiopancreatography (ERCP)-induced pancreatitis [22,24,25]. Studies show that people under age 35 who use cannabis recreationally or for certain medical reasons are at increased risk for pancreatitis [22,23,26,27]. Although most studies show an increased overall incidence of AP with cannabis use, some other studies have documented improved outcomes in AP patients using cannabis when compared to non-users. This is likely related to cannabis's anti-inflammatory response in the management of acute pancreatitis. In 2017, a clinical study done by Watanabe SM et al. found that patients using cannabis had a less severe form of pancreatitis than patients who were not using cannabis [20]. A similar study done in 2019 by Simons-Linares CR, Barkin JA, and Jang S et al. on in-hospital patients admitted with acute pancreatitis showed that cannabis use was associated with reduced mortality, morbidity, and costs of hospitalization in patients using cannabis compared to patients without it [25]. Despite various case reports and studies done on cannabis-induced pancreatitis, more studies are needed to support these findings and increase awareness of cannabis as a potential differential diagnosis of idiopathic acute pancreatitis.

Peptic ulcer disease

A gastro-duodenal ulcer is a rare complication of cannabis use, with limited studies to support its association. Cannabis exerts its effects on gastric secretion and emptying, and chronic cannabis use can slow down the healing process for gastroduodenal ulcers, leading to further complications [18]. Studies have shown that chronic cannabis use increases the likelihood of complications and hospitalization for peptic ulcer disease. A cross-sectional study done by Joundi H., Pereira KN, Haneef G. et al. on an inpatient data sample found that, compared to patients with peptic ulcer disease who do not use marijuana, patients using marijuana are 18 times more likely to get hospitalized for PUD [23,24].

Although no specific mechanism explains how cannabis slows down the healing process, a study done by Manzanares J., Julian M., and Carrascosa postulates that this effect has to do with the interaction of cannabis products on the endocannabinoid system (ECS). The ECS produces endocannabinoids which help to regulate pain and appetite and is composed of three main components: endocannabinoids, cannabinoid receptors, and enzymes that break down endocannabinoids [19]. The cannabinoids found in cannabis can interfere with the endocannabinoid system receptors, slow down the healing process, and increase the risk of complications [19].

Cannabis contains over 100 phytocannabinoids, including delta-9-tetrahydrocannabinol (THC) and cannabidiol (CBD), which interact with the ECS in various ways. THC binds to CBR1 receptors in the brain, which leads to the psychoactive effects associated with cannabis use. CBD, on the other hand, does not directly bind to cannabinoid receptors but instead modulates their activity through indirect mechanisms [2,7].

Regular cannabis use can interfere with the ECS by altering the expression of cannabinoid receptors and enzymes involved in endocannabinoid metabolism. Chronic exposure to THC, for example, can lead to downregulation of CBR1 receptors, which can result in a reduced ability of the ECS to modulate neurotransmitter release. Additionally, cannabis use can lead to changes in the levels of endocannabinoids in the body, which can also have downstream effects on ECS signaling [21,28].

Another study done by Chen Y, Zhang Y, Zhao Y, et al. on delayed wound healing in rats hypothesized this was due to the immunosuppressive effects of THC, which may have impaired the immune response necessary for wound healing [20]. Similarly, a study done by Zhang P., Huang Y., Hao H., et al. found that THC inhibited the migration of keratinocytes, which are the cells responsible for closing wounds. This study also found that THC decreased the production of collagen, which is necessary for wound healing and tissue repair [26].

The use of cannabinoids can also interfere with medications used to treat gastroduodenal ulcers, such as proton pump inhibitors and H2 receptor inhibitors. This can reduce the effectiveness of these drugs and increase the risk of complications. Cannabis use can also cause symptoms like those of gastroduodenal ulcers, such as abdominal pain and nausea. This can delay the diagnosis and treatment of underlying conditions, increasing the risk of complications [9,11,27].

It is important to note that more research is needed to fully understand the relationship between marijuana use and peptic ulcers and to determine to what extent other factors may contribute to this association. Overall, the effects of cannabis on wound healing are complex and depend on various factors, including the route of administration, dose, and duration of use. While some studies suggest that cannabis use can impair wound healing, more research is needed to fully understand the mechanisms involved.

## Conclusions

Cannabis, like every other substance abused, is linked to various GI diseases among the US adult population. It remains an issue of public health concern and calls for the awareness of medical professionals on the association between cannabis use and GI diseases, particularly GERD, Gastric ulcers, and Pancreatitis. There is no gainsaying that cannabis use and administration have their pros and cons (i.e., positive and negative effects) on the GI system. What remains, however, is to reveal to what extent positivity or negativity lies in the use of cannabis in medical practice. There is a need to uncover the actual causal relationship that exists between the use of cannabis and GI problems. This will help physicians get a better understanding of weighing risks and benefits with the use of cannabis and consequently strengthen precautionary measures, which are essential in the therapeutic use of cannabis in patient care.
